# SG-APSIC1031: Reappraisal of the effectiveness of a care bundle for patients with candidemia

**DOI:** 10.1017/ash.2023.67

**Published:** 2023-03-16

**Authors:** Changhua Chen, Yu-Min Chen

**Affiliations:** Infection Control, Taiwan; Changhua Christian Hospital, Changhua, Taiwan

## Abstract

**Objectives:** Candidemia has become one of the leading causes of healthcare-associated bloodstream infection, particularly in the intensive care unit. The management of candidemia remains challenging. We reassessed the protective effectiveness of a comprehensive care bundle on the management of candidemia and the effects of compliance with each element on the outcomes of patients. **Methods:** This network meta-analysis was conducted using the frequentist method. The participants included adult patients both infected with candidemia and who received bundle care. The primary outcome was the all-cause mortality among the patients included. **Results:** Studies in which a care bundle was created for patients with candidemia were identified, and 5 eligible studies with 5,808 participants were enrolled for further analysis. The random-effects model of the overall odds ratio (OR) revealed a significant reduction in the risk of all-cause mortality compared with that of the controls (OR, 0.599; 95% CI, 0.378–0.949; *P* = .025), as well as a reduction in the risk of developing persistent candidemia compared with the controls (OR, 0.483; 95% CI, 0.245–0.952; *P* = .008). In addition, no single element reached a protective effectiveness to improve the clinical outcome. **Conclusions:** This meta-analysis demonstrated that the combination of core elements in the care bundle resulted in protective effects, in that the all-cause mortality rates and incidence rates were effectively reduced among patients with persistent candidemia.

